# Activity and Habitat Use of Chimpanzees (*Pan troglodytes verus*) in the Anthropogenic Landscape of Bossou, Guinea, West Africa

**DOI:** 10.1007/s10764-016-9947-4

**Published:** 2017-01-30

**Authors:** Nicola Bryson-Morrison, Joseph Tzanopoulos, Tetsuro Matsuzawa, Tatyana Humle

**Affiliations:** 1 School of Anthropology and Conservation, University of Kent, Canterbury, Kent CT2 7NR UK; 2 Durrell Institute of Conservation and Ecology, School of Anthropology and Conservation, University of Kent, Canterbury, Kent CT2 7NR UK; 3 Kent’s Interdisciplinary Centre for Spatial Studies (KISS), University of Kent, Canterbury, Kent CT2 7NR UK; 4 Primate Research Institute, Kyoto University, Inuyama, Aichi 484-8506 Japan

**Keywords:** Forest–agricultural mosaic, Habitat selection, Human–wildlife coexistence, Risk perception

## Abstract

Many primate populations inhabit anthropogenic landscapes. Understanding their long-term ability to persist in such environments and associated real and perceived risks for both primates and people is essential for effective conservation planning. Primates in forest–agricultural mosaics often consume cultivars to supplement their diet, leading to potentially negative encounters with farmers. When crossing roads, primates also face the risk of encounters with people and collision with vehicles. Chimpanzees (*Pan troglodytes verus*) in Bossou, Guinea, West Africa, face such risks regularly. In this study, we aimed to examine their activity budget across habitat types and the influence of anthropogenic risks associated with cultivated fields, roads, and paths on their foraging behavior in noncultivated habitat. We conducted 6-h morning or afternoon follows daily from April 2012 to March 2013. Chimpanzees preferentially used forest habitat types for traveling and resting and highly disturbed habitat types for socializing. Wild fruit and crop availability influenced seasonal habitat use for foraging. Overall, chimpanzees preferred mature forest for all activities. They showed a significant preference for foraging at >200 m from cultivated fields compared to 0–100 m and 101–200 m, with no effect of habitat type or season, suggesting an influence of associated risk. Nevertheless, the chimpanzees did not actively avoid foraging close to roads and paths. Our study reveals chimpanzee reliance on different habitat types and the influence of human-induced pressures on their activities. Such information is critical for the establishment of effective land use management strategies in anthropogenic landscapes.

**Electronic supplementary material:**

The online version of this article (doi:10.1007/s10764-016-9947-4) contains supplementary material, which is available to authorized users.

## Introduction

Habitat loss due to deforestation and land conversion are major causes of the decline of nonhuman primate (hereafter primate) species (Chapman and Peres 2001; Estrada 2013). The continued degradation of forested areas, together with ongoing human population growth across most primate range countries, means that many primate populations now occur in forest–agricultural mosaics (Estrada 2013). Primates inhabiting these landscapes face multiple challenges including habitat degradation and fragmentation, human infrastructures such as roads or settlements, and increased encounters with people (Hockings *et al*. 2015). Their long-term survival critically depends on their ability to adapt to these human-dominated environments (Isabirye-Basuta and Lwanga 2008), as well as people’s tolerance of and behavior toward primates within these landscapes (Hill and Webber 2010).

Recent studies have revealed that many primates prefer areas with lower disturbance levels [chimpanzees (*Pan troglodytes*) and sooty mangabeys (*Cercocebus atys*): Brncic *et al*. 2015; bonobos (*Pan paniscus*): Hickey *et al*. 2013; chimpanzees, bonobos, and gorillas (*Gorilla* spp.): Junker *et al*. 2012; chimpanzees: Plumptre *et al*. 2010; mountain gorillas (*G. beringei beringei*): van Gils and Kayijamahe 2010; orangutans (*Pongo pygmaeus*): Wich *et al*. 2012]. These broad-scale studies have yielded important insights into the factors that influence the spatial distribution of a species on a national or regional scale. However, species persistence across landscapes can be scale dependent (Sawyer and Brashares 2013), and a finer-scale approach is required for understanding the effects of anthropogenic influences and disturbances on primate habitat use and behavioral flexibility (Bortolamiol *et al*. 2016). Such studies can help to inform land use planning aimed at balancing species conservation and development at a local scale in human-dominated environments.

Primate species show variable and multiple responses to environmental disturbances. Human-induced modifications in habitat quality can cause changes in primate feeding behavior, dietary diversity and resource use (Campbell-Smith *et al*. 2011; Guzmán *et al*. 2016; Lee 1997; Ménard *et al*. 2014; Pozo-Montuy *et al*. 2013; Riley 2007; Singh *et al*. 2001; Tutin 1999; Wong *et al*. 2006). Primate responses to the availability of wild and anthropogenic food sources are often species and/or context specific (McLennan and Hockings 2014). Some primates predominantly use areas of their home range in locations where important wild resources still remain (Heiduck 2002; Leighton 1993; Li 2004; O’Brien and Kinnaird 1997; Raboy and Dietz 2004; Riley 2008; Terada *et al*. 2015; Tweheyo *et al*. 2004). However, highly clumped and predictable food resources, such as exotic vegetation, cultivars, and human food waste, can also attract primates (Bortolamiol *et al*. 2016; Duvall 2008; Hill 2005; Hockings *et al*. 2009; Hoffman and O’Riain 2011; McKinney 2011).

Changes in primate habitat use, ranging, and activity budgets are often associated with anthropogenically disturbed environments. In locations where habitat quality and food resource availability are diminished, primates tend to exhibit larger home ranges and daily path lengths and spend more time traveling and less time resting and feeding, e.g., white-faced capuchins (*Cebus capucinus*: McKinney 2011) and long-tailed macaques (*Macaca fascicularis*: Sha and Hanya 2013). Conversely, primates that have access to, and use, spatially and temporally abundant human food sources tend to have smaller home ranges, spend less time traveling and foraging, and more time resting, e.g., yellow baboons (*Papio cynocephalus*: Altmann and Muruthi 1988), ring-tailed lemurs (*Lemur catta*: Gabriel 2013), and vervets (*Chlorocebus pygerythrus*: Saj *et al*. 1999). Most studies to date have focused on how habitat quality affects general patterns of primate activity budget allocation (Gabriel 2013; Guzmán *et al*. 2016; McKinney 2011; Riley 2007, 2008), while only a few have examined nonforaging activities across available habitat types within a landscape and within a single group (Terada *et al*. 2015). The preferences primates show for allocating activities to different habitats can provide insights into the relative value of these habitats, as well as species’ ability to adapt to habitat change (Palminteri and Peres 2012; Porter *et al*. 2007).

Risk and risk perception can also influence primate activity and range use. For example, predation risk influenced the use of different habitat types by chacma baboons (*Papio ursinus*) for resting and grooming (Cowlishaw 1997). Many primate species use their ranges strategically to offset the risk of predation with food acquisition (Hill 2016). Feeding is a risky behavior, and where individuals choose to feed can impact fitness and survival as much as what they choose to feed on (Lambert and Rothman 2015). It is likely that primates inhabiting anthropogenic landscapes aim to use habitats in such a way as to balance nutritional requirements with avoiding potential risks associated with human-induced pressures. Such risks can include negative interactions between farmers and primates due to cultivar foraging (Brncic *et al*. 2010; Hill 2000; Hockings *et al*. 2009; Hockings and Sousa 2013; McLennan 2013; Tweheyo *et al*. 2005), hunting pressure (Blake *et al*. 2007; Poulsen *et al*. 2009; Robinson *et al*. 1999), and risks from collisions with vehicles during road crossing (Cibot *et al*. 2015; McLennan and Asiimwe 2016). Chimpanzees, in particular, show a variety of adaptive behaviors in response to perceived risks associated with anthropogenic environments (Hockings *et al*. 2015), many of which have been likened to predator avoidance strategies (Hockings *et al*. 2006; Sakura 1994; Takemoto 2002). When foraging on cultivars, chimpanzees may increase group cohesiveness and vigilance behaviors (Hockings *et al*. 2007, 2012), vocalize less (Wilson *et al*. 2007), and forage at night to reduce the risk of detection by farmers (Krief *et al*. 2014). Chimpanzees also adapt their grouping patterns and behavior before and during road crossings (Cibot *et al*. 2015; Hockings 2011). Recent studies have demonstrated that primates display signs of anxiety and stress when faced with anthropogenic pressures (chimpanzees: Hicks *et al*. 2012; Hockings 2011; Hockings *et al*. 2006 and mountain gorillas: Muyambi 2005); some populations also show an increase in cortisol, a hormone that is released to buffer individuals in the short term from the effects of acute stress (Cyr and Romero 2008; Wingfield and Romero 2010), concentration levels [vervets: Fourie *et al*. 2015; spider monkeys (*Ateles geoffroyi yucatanensis*): Rangel‐Negrín *et al*. 2009]. Prolonged exposure to increased levels of anxiety and stress has negative impacts on fitness (Sapolsky *et al*. 2000). However, besides cultivar foraging and road crossing, we have a limited understanding of how human-induced pressures and risks impact primate habitat use and activity in anthropogenic landscapes.

The chimpanzee (*Pan troglodytes verus*) community at Bossou in Guinea, West Africa, is particularly well suited for examining responses to human disturbances and pressures. It has been rated as the most heavily impacted long-term chimpanzee research site (Wilson *et al*. 2014) and many aspects of chimpanzee ecology and behavior, as well as the practices and cultural beliefs of the local people, are well understood (Matsuzawa *et al.* 2011). Local people practice slash-and-burn agriculture, which has resulted in a highly heterogeneous anthropogenic landscape (Hockings *et al*. 2009; Sugiyama and Koman 1992). The density and availability of chimpanzee wild foods vary across forest and anthropogenic habitat types (Bryson-Morrison *et al*. 2016), and wild fruit availability is highly seasonal (Bryson-Morrison *et al*. 2016; Hockings *et al*. 2009; Takemoto 2002; Yamakoshi 1998). The chimpanzees regularly visit cultivated areas to forage on crops and cultivated fruit trees, particularly during seasonal wild fruit scarcity, although they consume some crops regardless of wild fruit availability (Hockings *et al*. 2009). The chimpanzees crop forage at any time of day, including on occasions when local people are present (Hockings 2007). The chimpanzees at this site are traditionally not hunted or killed because of the totemic beliefs of the local Manon people (Kortlandt 1986; Yamakoshi 2011). However, chimpanzee incursions into cultivated fields are rarely tolerated, and farmers frequently chase them away using noise and/or by throwing stones (Hockings *et al*. 2009). Two roads dissect the chimpanzees’ home range and crossing both these roads is necessary, but risky for them because of the high presence of vehicles and pedestrians (Hockings 2011). In response to these human-induced risks, Bossou chimpanzees display adaptive behaviors and increased frequencies of external signs of anxiety, i.e., rough-self scratching, when foraging in cultivated fields and crossing roads (Hockings 2011; Hockings *et al*. 2006, 2012).

We aimed to 1) determine Bossou chimpanzees’ overall and seasonal patterns of habitat use within their core area with respect to foraging, traveling, resting, and socializing and 2) examine the influences of risky areas, i.e., cultivated fields and human-made roads and paths, on foraging in noncultivated habitat. Given the highly seasonal availability of wild fruits coupled with the chimpanzees’ reliance on terrestrial herbaceous vegetation (THV) and cultivars, we predicted that chimpanzee use of forest and highly disturbed habitat types for foraging would reflect the spatial and temporal availability of food resources (Bryson-Morrison *et al*. 2016; Hockings *et al*. 2009; Takemoto 2002; Yamakoshi 1998). However, owing to the potential risks associated with encountering local people (Hockings 2011; Hockings *et al*. 2006, 2012), we also predicted that the chimpanzees would prefer habitat types with fewer human-induced pressures and, when foraging in noncultivated habitats, would avoid foraging close to cultivated fields and roads and paths (Cibot *et al*. 2015; Hockings 2011; Hockings *et al*. 2006, 2012).

## Methods

### Study Site and Population

We conducted our study in the anthropogenic landscape that surrounds the village of Bossou in the southeastern forest region of the Republic of Guinea, West Africa (latitude 7°38′71.7′N and longitude 8°29′38.9′W). Bossou is isolated from the nearest stretch of continuous mature forest in the Nimba Mountain range by *ca*. 6 km of savannah. The climate in this region is classified as tropical wet seasonal (Richards 1996), with a short dry season from November to February, when wild fruit availability is highest, and a distinct rainy season from March to October, when wild fruit availability is lower (Bryson-Morrison *et al*. 2016; Hockings *et al*. 2009; Humle 2011; Takemoto 2002; Yamakoshi 1998). Four small hills (70–150 m high) surround the village of Bossou and form the core area (*ca*. 6 km^2^) of the resident chimpanzee community that ranges in this landscape (15 km^2^ home range) (Humle 2011). During our study (April 2012–March 2013), the chimpanzee community size ranged between 12 and 13 individuals, with 4 adult males and 6 adult females. The Bossou chimpanzees exhibit less fission–fusion than other known communities (Hockings *et al*. 2012), often traveling and foraging in larger parties than expected relative to community size (Matsuzawa *et al*. 2011).

### Habitat Composition and Food Availability

We determined habitat composition using quadrat sampling that covered >70% (4.3 km^2^) of the chimpanzees’ core area, excluding village areas, roads and paths, and rivers (Bryson-Morrison *et al*. 2016). Regenerating forest, i.e., young and older growth secondary forest, dominates the landscape, although areas of riverine forest and one small patch of mature forest remain (Bryson-Morrison *et al*. 2016; Humle 2011). Cultivated fields, coffee plantations, and fallow areas, of various successional stages, occur throughout (Bryson-Morrison *et al*. 2016; Humle 2011). We included all forest, i.e., mature, riverine, secondary, and young secondary forest, and highly disturbed, i.e., fallow stage 1, 2, and 3; coffee plantations; and cultivated fields, habitat types in our study (Table I) (Bryson-Morrison *et al*. 2016).

**Table I:** Description of the habitat types available at Bossou, Guinea, including percentage availability in the chimpanzees’ core area, stem density/ha of food tree species (≥10 cm DBH), terrestrial herbaceous vegetation (THV) density/m^2^, and percentage chimpanzee foraging time

Habitat types	Description	% availability	Stem density/ ha	THV density/ m^2^	% foraging time
Forest					
Mature forest	Old growth forest >70 year old. Concentrated on the summit of one of the small hills, known as Gban. Dense forest with little to no signs of human disturbance.	4	250	3.0	15
Riverine forest	Seasonally flooded forest, located along waterways with an approximate width of 20 m on either side.	8	150	3.3	4
Secondary forest	Mature secondary regrowth of vegetation. 30+ years old with a closed canopy.	25	248	1.3	23
Young secondary forest	Young secondary regrowth of vegetation. > 15 years old with an open canopy. Dominated by small, young regenerating tree species.	15	306	0.4	6
Highly disturbed					
Fallow stage 1	Cultivated areas abandoned <1 year ago. Cultivars still present. Dominated by an invasive species, *Chromolaena odorata*.	8	56	0.3	2
Fallow stage 2	*Chromolaena odorata* still present but no longer dominant. Tree saplings, lianas, and THV emerging.	8	28	0.4	8
Fallow stage 3	*Chromolaena odorata* no longer present, <15 years old. Characterized by dense tree saplings, lianas, and THV.	15	100	2.3	11
Coffee plantation	Maintained areas dominated by cultivated coffee trees. Banana plants, oil palm, and cultivated fruit tree orchards often present.	9	45	0.2	17
Cultivated field	Characterized by active cultivation. Usually contains a mix of cultivars such as cassava, maize, okra, and rice.	9	80	0	14

Forest habitats age categories were adapted from Schroeder *et al*. (2010) and Sugiyama and Koman (1979, 1992). The ecological characteristics of these habitat types are provided in further detail in Bryson-Morrison *et al*. (2016).

**Fig. 1:**
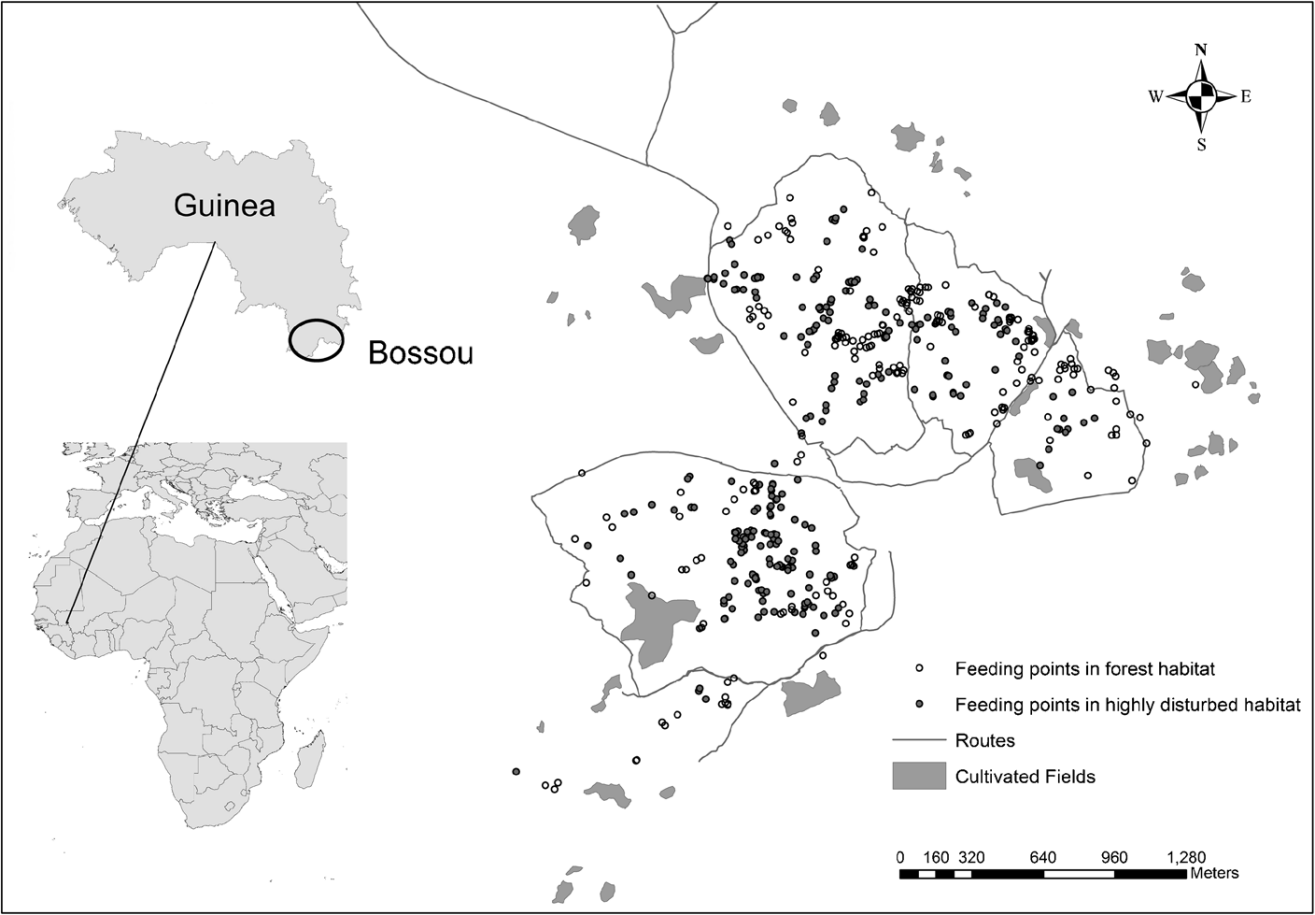
Map showing the location of all chimpanzee feeding event points (*N* = 474) in forest habitat (mature, riverine, secondary, and young secondary forest) (open circles) and highly disturbed habitat (fallow stage 1, 2, and 3 and coffee plantations) (closed circles) in relation to cultivated fields and routes (all human-made roads and paths) across the chimpanzee core area in Bossou, Guinea, West Africa. We collected feeding event points from April 2012 to March 2013.

Regenerating and mature forest contain the highest densities of chimpanzee food tree species, while highly disturbed habitat types show relatively low densities (Table I) (Bryson-Morrison *et al*. 2016). THV occurs in high densities in most forest habitat types, and in fallow stage 3 areas (Table I) (Bryson-Morrison *et al*. 2016; Humle 2011) and is found at relatively low densities in all other highly disturbed habitat types (Table I) (Bryson-Morrison *et al*. 2016). The majority of cultivated fields in Bossou contain a mix of crops including maize (*Zea mays*), cassava (*Manihot esculenta*), okra (*Hibiscus esculentus*), rice (*Oryza* sp.), banana (*Musa sinensis*), and pineapple (*Ananasa comosus*), all of which provide food parts that are consumed by the chimpanzees (Hockings *et al*. 2009). In addition to coffee trees (*Coffea* sp.), most coffee plantations in Bossou contain cultivated fruit tree orchards that provide fruits consumed by the chimpanzees such as orange (*Citrus sinensis*), mandarin (*Citrus reticulata*), mango (*Mangifera indica*), and cacao (*Theobroma cacao*), as well as banana plants. Unlike cultivated fields, coffee plantations are seldom guarded and the chimpanzees are rarely chased away even when local people are present (Bryson-Morrison *pers. obs*.). Human-made roads and paths (routes) are found throughout the chimpanzees’ home range (Fig. 1). The larger of the two dirt roads (*ca*. 12 m wide) serves as a main thoroughfare from Liberia to the forest region of Guinea and is frequently used by vehicles and pedestrians (Hockings 2011). The smaller road (*ca*. 3 m wide) runs to nearby villages and is used by pedestrians and motorcycles (Hockings 2011). Small paths dissect all four hills and are used by local people for access to forest and agricultural areas.

### Mapping of Anthropogenic Features

We mapped routes (2 roads, 7 paths) and cultivated fields (43 fields) in the Bossou chimpanzees’ core area using a handheld Garmin 62S GPS set to record a point every 10 m (open canopy accuracy of ± 3 m for GPS points) (Fig. 1).

### Behavioral Observations and Feeding Event Locations

We collected data over a 12-months period from April 2012 to March 2013. We conducted behavioral follows for a maximum of 6 h/day to comply with site regulations aimed at limiting the time spent observing the chimpanzees. We conducted behavioral follows in the morning between 06:30 and 12:30 h (*N* = 331 h) or afternoon between 12:30 and 18:30 h (*N* = 237 h) [total observations: 568 h; wet season (March–October): 440 h; dry season (November–February): 128 h]. We began daily follows when we first encountered the chimpanzees. Before each daily follow, we randomly selected an adult focal individual from a predetermined list to record all feeding events using a handheld Garmin 62S GPS. We sampled all adult individuals (*N* = 10) at least once per month. We defined a feeding event as foraging on a single food type and plant part from the same individual tree or food patch. We also recorded habitat type for all feeding events (Table I). Feeding events excluded foraging on crops in cultivated fields, as we avoided following the chimpanzees into these areas during cultivar foraging during our study to minimize the risk that our presence would be viewed negatively by farmers. We observed the chimpanzees from a distance whenever possible to determine their activities within fields; however, this means that we may have underestimated chimpanzee use of cultivated fields. We used focal feeding event points (forest habitat: *N* = 269; highly disturbed habitat: *N* = 205) in spatial analyses to examine the distance from feeding events in noncultivated habitat to cultivated fields and routes. We also conducted 15-min instantaneous scan sampling (Altmann 1974) to record habitat type and activity, i.e., traveling, resting, socializing, and foraging (including actively searching, consuming, and handling food items), for all individuals present in the focal individual’s party (mean party size: 6.8 ± 0.6) (Lehmann *et al*. 2007). We performed all analyses at the community level because of the small size of the Bossou chimpanzee community at the time of this study.

### Data Analyses

#### Habitat Use and Preferences

To examine chimpanzee habitat selection, we summed the number of 15-min scans in forest habitat, i.e., mature, riverine, secondary, and young secondary forest combined, and highly disturbed habitat, i.e., fallow stage 1, 2, and 3; coffee plantations; and cultivated fields combined, for the entire research period (12 months) and for the wet and dry seasons. We also quantified habitat selection for each of the four mutually exclusive activities (foraging, traveling, resting, and socializing). We then examined habitat selection for each individual habitat type for all activities. Following Manly *et al*. (2002), we used a Pearson chi-square test to examine the null hypothesis that chimpanzee habitat selection was proportional to habitat availability. Similarly, we used a chi-square test to examine the null hypothesis that chimpanzee activities in each habitat type were proportional to the total number of observations. The results of both the chi-square tests allowed us to examine whether the chimpanzees were selectively using or avoiding a particular habitat type by calculating selection ratios using the following equation:

$$ W i=\frac{Oi}{\pi i} $$

where $O i$ is the proportion of observations in habitat type *i* to the total number of recorded observations and $\pi i$ is the proportion of area comprising habitat type *i* to the entire area available (Manly *et al*. 2002). $W i$ values >1 indicate a positive selection for habitat type *i*, values <1 indicate a negative selection for habitat type *i*, and values around 1 indicate that habitat type *i* was used proportionally to its availability. We standardized selection ratios to allow comparisons between studies using Manly’s standardized selection ratio (Manly *et al*. 2002):

$$ Bi=\frac{Wi}{{\displaystyle {\sum}_{j=1}} Wj} $$

Manly’s standardized selection ratio ranges from 0 (no observations in a habitat) to 1 (all observations in a habitat) and provides a measure of the estimated probability that habitat type *i* would be the next one selected if all habitat types were equally available (Manly *et al*. 2002). We considered habitat types with the highest selectivity index (*Bi*) for each activity as preferred habitat for the chimpanzees. We then examined if habitat selection ratios were statistically significant using the following equation:

$$ {X}^2={\left\{\frac{Wi-1}{\mathrm{SE}(Wi)}\right\}}^2 $$

where $\mathrm{S}\mathrm{E}(Wi)$ is the standard error of the selection ratio for habitat type *i* (Manly *et al*. 2002). We further compared if selection ratios for each habitat type were significantly different from each other using the following equation:

$$ {X}^2=\frac{{\left( Wi- Wj\right)}^2}{\mathrm{var}\left( Wi- Wj\right)} $$

where $\mathrm{v}\mathrm{a}\mathrm{r}\left( Wi- Wj\right)$ is the variance of the difference between the selection ratios for habitat type *i* and *j* (Manly *et al*. 2002). For all chi-square tests, we applied a *Z*-test with Bonferroni adjusted 95% confidence intervals of the standardized residuals (Byers *et al*. 1984; Manly *et al*. 2002; Neu *et al*. 1974).

#### Distance of Feeding Events Relative to Cultivated Fields and Routes

We used QGis 2.14.0-Essen to calculate the nearest distance (m) of each chimpanzee feeding event point (*N* = 474) to cultivated fields (range: 5.1–681.5 m; mean distance = 352.87 ± 8.29 m) and routes (range: 1.0–593.8 m; mean distance = 170.01 ± 5.24 m) for the full year and for the wet and dry seasons (Fig. 1). We grouped the distance from feeding event points to cultivated fields and routes into 0–100 m, 101–200 m, >200 m categories to facilitate analyses (*sensu* Lehman *et al*. 2006). We used a Pearson chi-square test to examine the null hypothesis that the frequency of chimpanzee feeding events was the same for all distance categories to cultivated fields and routes. We then examined the influence of habitat type and season on feeding event distance to cultivated fields and routes using a two-way ANOVA. To meet the assumptions for Levene’s test for equality of variance and normality distribution of the data, we removed three outliers and square root transformed the feeding event point distances to routes, and cube transformed the feeding event point distances to cultivated fields. We carried out all statistical analyses using SPSS v. 22 and set the significance level at *P* ≤ 0.05.

## Ethical Note

Our study adhered to all research requirements of Guinea, the ethical protocols for research set out by the University of Kent, UK and the Kyoto University Primate Research Institute, Japan, the institution that manages the Bossou fieldsite. The authors have no conflict of interest or competing financial interests to decla﻿re﻿.

## Results

### Habitat Use and Preferences

#### Patterns of Overall Habitat Use

**Fig. 2:**
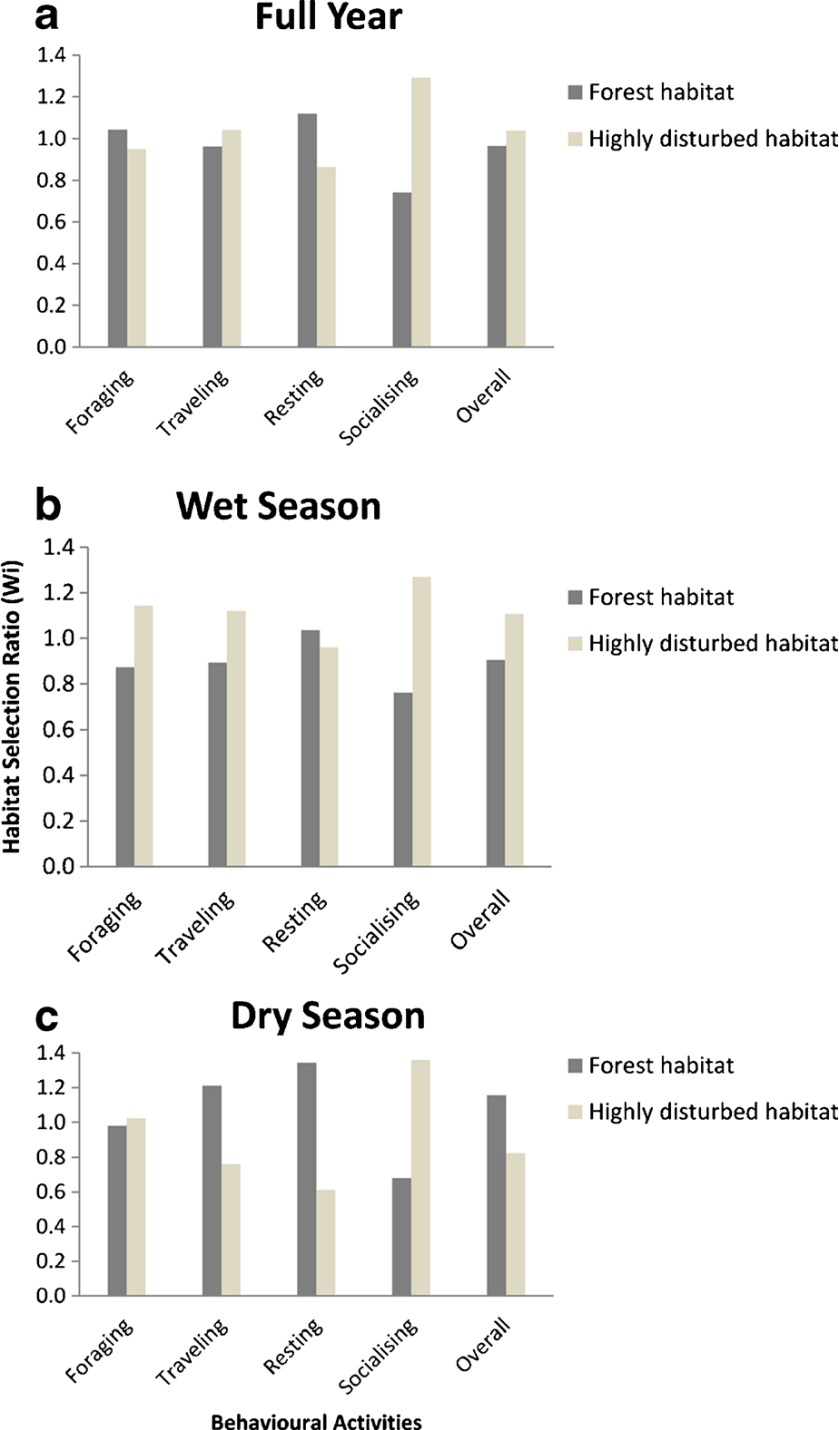
Habitat selection ratios (*Wi*) (Manly *et al*. 2002) for four activities and overall (aggregate of 15-min scans for each habitat type) for the chimpanzee community at Bossou, Guinea, West Africa. **a** Full year (April 2012–March 2013). **b** Wet season (March–October). **c** Dry season (November–February). Forest habitat (mature, riverine, secondary, and young secondary forest) and highly disturbed habitat (fallow stage 1, 2, and 3; coffee plantations; and cultivated fields).

Habitat selection ratio (*Wi*) values for the full year for all chimpanzee activities were similar for forest habitat (mature, riverine, secondary, and young secondary forest combined) (*Wi* = 0.74–1.04) and highly disturbed habitat (cultivated fields; coffee plantations; and fallow stages 1, 2, and 3 combined) (*Wi* = 0.86–1.29) (Fig. 2a). Selection ratio values for the wet season suggested that the chimpanzees used highly disturbed habitat marginally more than forest habitat for all activities other than resting (forest habitat range *Wi* = 0.76–0.91; highly disturbed *Wi* = 0.96–1.27) (Fig. 2b). However, during the dry season, the chimpanzees used forest habitat more for resting and traveling and overall (all activities combined) and used highly disturbed habitat more for socializing (Fig. 2c).

When we examined the Bonferroni adjusted standardized residuals (*χ* ^2^ tests) for individual habitat types, selection ratios were significantly different between all habitat types, except between young secondary forest and fallow stage 1, and between fallow stage 3 and coffee plantations. Furthermore, selection ratios were significantly different for each of the four activities and overall for all habitat types and all time periods, with the exception of foraging, resting, and traveling in fallow stage 1 during the dry season (Table II and Electronic Supplementary Material Table SI). Overall, mature forest emerged as the most preferred habitat for the chimpanzees with the highest standardized selection ratios (*Bi*) during all time periods (overall: wet season: *Bi* = 0.22; dry season: *Bi* = 0.46; full year: *Bi* = 0.29). Generally, fallow stage 1 was the least preferred habitat type for the chimpanzees for all activities and time periods (overall: wet season: *Bi* = 0.04; dry season: *Bi* = 0.02; full year: *Bi* = 0.04), followed closely by young secondary forest (overall: wet season: *Bi* = 0.04; dry season: *Bi* = 0.02; full year: *Bi* = 0.04) (Table SI).

**Table II:**
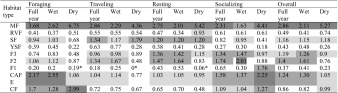
Chimpanzee habitat selection ratios (*Wi*) (Manly *et al*. 2002) for each habitat type at Bossou, Guinea, during the wet season (March–October), dry season (November–February), and full year (April 2012–March 2013) for four activities and overall (aggregate of 15-min scans)

* Denotes selection ratios that were not significant. Selection ratios highlighted in dark-gray: *Wi* ≥ 2.00: highly preferred; mid-gray: *Wi* = 1.20–1.99: preferred; light-gray: *Wi* = 0.90–1.19: used proportionally to availability; unshaded: *Wi* = 0–0.89: avoided. Forest habitat: MF: mature forest; RVF: riverine forest; SF: secondary forest; YSF: young secondary forest; Highly-disturbed habitat: F3: fallow stage 3; F2: fallow stage 2; F1: fallow stage 1; CAFE: coffee plantation; CF: cultivated field

#### Habitat Preference for Foraging

For the forest habitat types and given relative habitat availability, selection ratios revealed that the chimpanzees highly preferred mature forest for foraging during all time periods. Generally, chimpanzees used secondary forest relative to its availability for foraging and avoided riverine and young secondary forest during all time periods. Of the highly disturbed habitat types, chimpanzees preferred coffee plantations and cultivated fields for foraging across the full year, with coffee plantations being highly preferred during the wet season and cultivated fields highly preferred during the dry season. Chimpanzees avoided all stages, i.e., 1, 2, and 3, of fallow habitat for foraging (Tables II and SI).

#### Habitat Preference for Other Activities. Traveling

Chimpanzees highly preferred mature forest for traveling, used secondary forest relative to availability, and avoided riverine and young secondary forest for traveling regardless of season. Generally, they used fallow stage 2 and 3 and coffee plantations relative to availability, except during the dry season, when they avoided these areas. Chimpanzees avoided fallow stage 1 and cultivated fields across all time periods (Tables II and SI).

#### Resting

The chimpanzees highly preferred mature forest for resting during all time periods. They used secondary forest relative to availability across all time periods, and used riverine forest relative to availability during the dry season but avoided it during the wet season and full year. They avoided young secondary forest across all time periods. Generally, the chimpanzees used coffee plantations and fallow stages 3 and stage 2 relative to availability for resting. They avoided fallow stage 1 and cultivated fields for resting during all time periods (Tables II and SI).

#### Socializing

Of the forest habitats, chimpanzees preferred only mature forest for socializing. They used secondary forest relative to availability during the wet season. Of the highly disturbed habitats, the chimpanzees generally preferred socializing in fallow stages 3 and stage 2 and coffee plantations. Generally, chimpanzees used cultivated fields relative to availability and preferred fallow stage 1 for socializing during the dry season but avoided it during the wet season and full year (Tables II and SI).

### Distance of Feeding Events in Noncultivated Habitat Relative to Cultivated Fields and Routes

There was a significant difference in feeding event distance categories to cultivated fields for the full year and both the wet and dry seasons (full year: *χ* ^2^ = 433.841, df = 2, *P* < 0.0001; wet season: *χ* ^2^ = 280.760, df = 2, *P* < 0.0001; dry season: *χ* ^2^ = 158.423, df = 2, *P* < 0.0001). Inspection of the standardized residuals revealed that the chimpanzees fed less than expected by chance at 0–100 m and 101–200 m and more than expected by chance >200 m away from cultivated fields during the wet and dry seasons and full year. We also found no effect of habitat type or season on feeding event distance to cultivated fields (two-way ANOVA, *F*(1, 467) = 0.430, *P* = 0.512).

There was no significant difference between the observed and expected values for chimpanzee feeding event distance to routes for the wet and dry seasons and full year (full year: *χ* ^2^ = 1.466, df = 2, *P* = 0.480; wet season: *χ* ^2^ = 1.031, df = 2, *P* = 0.597; dry season: *χ* ^2^ = 0.437, df = 2, *P* = 0.804). However, the two-way ANOVA revealed a statistically significant interaction between habitat type and season on the distance of feeding events to routes (*F*(1, 467) = 5.227, *P* = 0.023). Specifically, the distance of feeding events to routes was greater during the wet season than the dry season in highly degraded habitat. However, there was no effect of season on feeding event distance to routes for forest habitat.

## Discussion

Our study revealed that the chimpanzee community inhabiting the highly heterogeneous anthropogenic landscape of Bossou used different habitat types with varying frequency depending on season and behavioral activity.

### Habitat Preference for Foraging

Our results support the prediction that chimpanzee patterns of habitat use for foraging reflect spatial and temporal food resource availability. Mature forest harbors high densities of chimpanzee food tree species and THV, and the chimpanzees preferentially used this habitat type for foraging throughout the year and especially during the dry season, when wild fruit availability was high (Bryson-Morrison *et al*. 2016). The chimpanzees also preferentially used cultivated fields for foraging during the dry season, which coincides with the availability of many crops (Hockings *et al*. 2009). Coffee plantations had the same selection ratio as mature forest during the wet season when wild fruit abundance was lower (Bryson-Morrison *et al*. 2016). Coffee plantations provide the chimpanzees with easily attainable spatially clumped fruit trees, many of which produce ripe fruit during the wet season, or year round (Bryson-Morrison *et al*. 2016; Hockings *et al*. 2009). Furthermore, the chimpanzees generally avoided fallow habitats, which have relatively low food availabilities (Table I) (Bryson-Morrison *et al*. 2016). Similarly to other chimpanzee communities, Bossou chimpanzees consume a diverse range of foods but maintain a high annual proportion of fruit in their diets (Hockings *et al*. 2009; Takemoto 2002; Yamakoshi 1998), which significantly influenced their habitat use and foraging strategies. These patterns are similar to those reported for other chimpanzee communities, e.g., Caiquene-Cadique, Cantanhez National Park, Guinea-Bissau (Bessa *et al*. 2015); Bafing Biosphere Reserve, Mali (Duvall 2008); Budongo, Uganda (Tweheyo *et al*. 2004); and Kahuzi, Democratic Republic of Congo (Basabose 2005). Our study reveals that Bossou chimpanzees specifically prefer mature forest year round for foraging, although they also rely heavily on agricultural habitat to supplement their diets with cultivars. As we did not record all incursions into cultivated fields, we may have underestimated the importance of this habitat type relative to other habitat types.

### Habitat Preference for Other Activities

Our results indicated that Bossou chimpanzees preferred to travel, rest, and socialize in habitat types with less human-induced pressure. Older growth forest (mature and secondary forest) offers greater tree cover (Bryson-Morrison *et al*. 2016) and little to no human presence, while cultivated fields are relatively open areas (Bryson-Morrison *et al*. 2016) with high human presence and a high likelihood of antagonistic interactions with humans (Hockings *et al*. 2007, 2009). Preferential use of mature forest in the dry season, when daily temperatures are high and precipitation low (Humle 2011), for all activities may also reflect an increased requirement for shade. The chimpanzees are known to display thermoregulatory behavior during the dry season by increasing terrestriality to take advantage of cooler temperatures on the ground compared to higher positions in the trees (Takemoto 2004). The high densities of the invasive shrub, *Chromolaena odorata*, which form dense thickets that are difficult to navigate through, may explain chimpanzees’ avoidance of stage 1 fallow (Bryson-Morrison *pers. obs*.). Nevertheless, our results show that the chimpanzees did not actively avoid all highly disturbed habitat types and used some preferentially, depending on activity and season. Although not examined in the context of specific nonforaging activity patterns, other ecologically flexible primates, such as macaques (Riley 2008) and baboons (Hoffman and O’Riain 2011), often preferentially use human-modified habitats. The high occurrence of social activity in coffee plantations and cultivated fields reflects increased group cohesiveness and social behavior previously reported for Bossou chimpanzees foraging on cultivars (Hockings *et al*. 2012). Consumption of nutritious energy-rich crops in cultivated areas may allow them more time to engage in other activities, such as socializing, as in populations of baboons, vervets, and macaques consuming human food sources (Altmann and Muruthi 1988; Brennan *et al*. 1985; Schlotterhausen 2000).

Bossou chimpanzees generally avoided riverine forest habitat. This pattern contrasts with findings from Bulindi, Uganda, where chimpanzees heavily use riverine forest fragments that contain a higher density of feeding trees than the Budongo Forest Reserve, the nearest main forest block (McLennan and Plumptre 2012). Several factors may explain this difference. The density of chimpanzee food tree species in riverine forest at Bossou is low compared to that in other forest types (Table I) (Bryson-Morrison *et al*. 2016). Second, riverine forest patches in Bossou are relatively small and often abut cultivated fields, and there is a higher human presence in these areas than within other noncultivated habitat types. This suggests that the availability of a particular habitat type is not necessarily a good indicator of use by chimpanzees, as habitat quality and perceived risks likely vary across sites.

Chimpanzee avoidance of young secondary forest is more difficult to interpret, particularly as this forest type harbors a high density of chimpanzee food species (Bryson-Morrison *et al*. 2016). The chimpanzees may be selecting older growth forests for feeding on wild fruits as larger trees are known to produce greater fruit yields (Chapman *et al*. 1992). More detailed phenological surveys of fruiting patterns between habitat types are needed to test this.

### Distance of Feeding Events to Cultivated Fields and Routes

Our results indicated that the chimpanzees significantly preferred foraging on foods in noncultivated habitat at >200 m compared to 0–100 m and 101–200 m from cultivated fields during all time periods, with no effect of habitat type or season. Wild fruit scarcity during the wet season and ease of access to cultivars did not appear to influence distance of feeding events to cultivated fields, contrasting with findings for the chimpanzee community at Sebitoli, Kibale National Park, Uganda (Bortolamiol *et al*. 2016). Instead our results suggest that the chimpanzees’ preference for foraging on foods in noncultivated habitat at a greater distance to cultivated fields was more likely driven by perceived risks associated with these areas (Hockings 2007, 2011). The nutritional benefits gained from acquiring wild foods close to cultivated fields may not be enough to offset any risks associated with potential human presence, as has been proposed for cultivar foraging (Hockings *et al*. 2009; McLennan and Hockings 2014; Naughton-Treves *et al*. 1998). The chimpanzees may therefore be using their environment strategically to balance food acquisition and risk avoidance (Hill 2016). Future studies should aim to collect more detailed phenological data on the availability of food resources at varying distances to cultivated fields, along with behavioral and/or cortisol measures of stress, to investigate fully the effects of risky areas on chimpanzee foraging behavior.

We found no significant difference in chimpanzee feeding event distance to routes (human-made roads and paths). However, the chimpanzees foraged in highly degraded habitat at a greater distance from routes during the wet season than the dry season with no such seasonal effect found for forest habitat. This suggests that the Bossou chimpanzees did not actively avoid foraging close to routes; instead, feeding event distance from routes was likely driven by food availability. Pioneer tree species that produce fruits consumed by the chimpanzees, including *Musanga cecropioides*, semidomesticated and wild oil palm (*Elaeis guineensis*), and coffee plantations containing fruit orchards and banana plants, are found at the sides of roads and paths (Bryson-Morrison *pers. obs*.). Road crossing is risky for wildlife, including primates (Cibot *et al*. 2015; Gunson *et al*. 2011; Jaegger *et al*. 2005; McLennan and Asiimwe 2016); however, roadsides can also represent areas of high vegetation species richness, attracting wildlife (Forman and Alexander 1998). Indeed, findings from Sebitoli, Kibale National Park, Uganda indicated that proximity to a tarmac road, where roadside management strategies favor the growth of THV, was one of the main predictors of chimpanzee distribution (Bortolamiol *et al*. 2016).

### Implications for Chimpanzee Conservation in Anthropogenic Landscapes

Overall, our study clearly indicated that chimpanzees at Bossou show a high preference for mature forest. Local people rarely gather nontimber forest products from, or enter, this single small patch of mature forest as they regard it as sacred (Kortlandt 1986; Yamakoshi 2005). We also found chimpanzees rarely use riverine forest at Bossou, probably because this combines relatively low food availability with high human presence. Our results suggest that chimpanzees in human-dominated environments prefer habitat types where a plentiful supply of wild foods is coupled with low human presence for most activities. The availability of such “refuges” may be critical to the long-term persistence of chimpanzee populations within anthropogenic landscapes.

Alongside older-growth forest (mature and secondary forest), the chimpanzees at Bossou preferentially used cultivated habitat for foraging throughout the year. Chimpanzee reliance on crops to supplement wild foods in forest–agricultural mosaics complicates human–chimpanzee coexistence and requires careful management (Hill and Wallace 2012). Restoration or recovery of abandoned agricultural areas to forest may reduce reliance on cultivated food, but this will likely depend on how important crops are in the diet of a given population, as well as the degree of perceived risk associated with cultivar foraging in agricultural habitats (Hockings and McLennan 2012; McLennan and Hockings 2014). Moreover, reforestation of abandoned agricultural areas can take many years (Aide *et al*. 2000; Chapman and Chapman 1999) and young successional habitat types may be the only available habitats for resident chimpanzees in the interim. Our study showed that chimpanzees generally avoided using young regenerating habitat types (fallow and young secondary forest), suggesting that widespread agricultural conversion and subsequent expansion of new fallow areas could prove detrimental for the long-term survival of chimpanzees, as for other primate populations (Ancrenaz *et al*. 2015; Palm *et al*. 2013; Wich *et al*. 2014).

In conclusion, our study reveals that the risks associated with some anthropogenic features may influence important behavioral activities, such as foraging. These findings contribute to our understanding of chimpanzee behavioral responses to human encounters and pressures in their environment. Our study further demonstrates the value of determining which habitat types are avoided or preferred, and potentially necessary, for chimpanzees in anthropogenic landscapes. We suggest that it is crucial to determine relative reliance on available habitat types, as well as agricultural areas, when devising conservation strategies for chimpanzee and other primate populations residing in anthropogenic landscapes.

## Electronic Supplementary Material

Below is the link to the electronic supplementary material.

ESM 1 (DOCX 44 kb)
