# Supplementary material for: Activity and Habitat Use of Chimpanzees (Pan troglodytes verus) in the Anthropogenic Landscape of Bossou, Guinea, West Africa
Source: Int J Primatol. 2017 Jan 30;38(2):282–302. doi: 10.1007/s10764-016-9947-4 (PMC5422491; doi:10.1007/s10764-016-9947-4)
Supplement: Supplementary file 1 — (DOCX 44 kb) [file 10764_2016_9947_MOESM1_ESM.docx]

**Electronic Supplementary Material**

**Activity and Habitat Use of Chimpanzees (*Pan troglodytes verus*) in the Anthropogenic Landscape of Bossou, Guinea, West Africa**

**Nicola Bryson-Morrison ∙ Joseph Tzanopoulos ∙ Tetsuro Matsuzawa ∙ Tatyana Humle**

**Table SI** Observed and expected chimpanzee habitat selection with selection ratios (*Wi*) and standardized selection ratios (*Bi*)

| Period | Behavior |  | MF | RF | SF | YSF | F3 | F2 | F1 | CAFE | CF |
| --- | --- | --- | --- | --- | --- | --- | --- | --- | --- | --- | --- |
| Full year | Foraging | Expected | 60.6 | 136.4 | 378.8 | 227.3 | 227.3 | 121.2 | 121.2 | 121.2 | 121.2 |
|  |  | Observed | 223 | 56 | 355 | 89 | 169 | 128 | 24 | 263 | 208 |
|  |  | *Wi* | 3.68 | 0.41 | 0.94 | 0.39 | 0.74 | 1.06 | 0.20 | 2.17 | 1.72 |
|  |  | *Bi* | 0.33 | 0.04 | 0.08 | 0.03 | 0.07 | 0.09 | 0.02 | 0.19 | 0.15 |
|  | Resting | Expected | 239.4 | 538.6 | 1496 | 897.6 | 897.6 | 478.7 | 478.7 | 478.7 | 478.7 |
|  |  | Observed | 658 | 253 | 1797 | 345 | 1221 | 702 | 205 | 492 | 311 |
|  |  | *Wi* | 2.75 | 0.47 | 1.20 | 0.38 | 1.36 | 1.47 | 0.43 | 1.03 | 0.65 |
|  |  | *Bi* | 0.28 | 0.05 | 0.12 | 0.04 | 0.14 | 0.15 | 0.04 | 0.11 | 0.07 |
|  | Traveling | Expected | 89.8 | 202.1 | 561.3 | 336.8 | 336.8 | 179.6 | 179.6 | 179.6 | 179.6 |
|  |  | Observed | 257 | 111 | 752 | 213 | 323 | 241 | 32 | 186 | 130 |
|  |  | *Wi* | 2.86 | 0.55 | 1.34 | 0.63 | 0.96 | 1.34 | 0.18 | 1.04 | 0.72 |
|  |  | *Bi* | 0.30 | 0.06 | 0.14 | 0.07 | 0.10 | 0.14 | 0.02 | 0.11 | 0.08 |
|  | Socializing | Expected | 42.0 | 94.6 | 262.8 | 157.7 | 157.7 | 84.1 | 84.1 | 84.1 | 84.1 |
|  |  | Observed | 97 | 58 | 215 | 43 | 212 | 146 | 55 | 133 | 92 |
|  |  | *Wi* | 2.31 | 0.61 | 0.82 | 0.27 | 1.34 | 1.74 | 0.65 | 1.58 | 1.09 |
|  |  | *Bi* | 0.22 | 0.06 | 0.08 | 0.03 | 0.13 | 0.17 | 0.06 | 0.15 | 0.10 |
|  | Overall | Expected | 431.8 | 971.55 | 2698.8 | 1619.3 | 1619.3 | 863.6 | 863.6 | 863.6 | 863.6 |
|  |  | Observed | 1235 | 478 | 3119 | 690 | 1925 | 1217 | 316 | 1074 | 741 |
|  |  | *Wi* | 2.86 | 0.49 | 1.16 | 0.43 | 1.19 | 1.41 | 0.37 | 1.24 | 0.86 |
|  |  | *Bi* | 0.29 | 0.05 | 0.12 | 0.04 | 0.12 | 0.14 | 0.04 | 0.12 | 0.09 |
| Wet | Foraging | Expected | 45.0 | 101.3 | 281.5 | 168.9 | 168.9 | 90.1 | 90.1 | 90.1 | 90.1 |
|  |  | Observed | 118 | 38 | 289 | 76 | 141 | 101 | 18 | 230 | 115 |
|  |  | *Wi* | 2.62 | 0.37 | 1.03 | 0.45 | 0.83 | 1.12 | 0.20 | 2.55 | 1.28 |
|  |  | *Bi* | 0.25 | 0.04 | 0.10 | 0.04 | 0.08 | 0.11 | 0.02 | 0.24 | 0.12 |
|  | Resting | Expected | 187.4 | 421.6 | 1171 | 702.6 | 702.6 | 374.7 | 374.7 | 374.7 | 374.7 |
|  |  | Observed | 376 | 144 | 1408 | 290 | 997 | 616 | 199 | 393 | 261 |
|  |  | *Wi* | 2.01 | 0.34 | 1.20 | 0.41 | 1.42 | 1.64 | 0.53 | 1.05 | 0.70 |
|  |  | *Bi* | 0.25 | 0.04 | 0.13 | 0.04 | 0.15 | 0.18 | 0.06 | 0.11 | 0.07 |
|  | Traveling | Expected | 65.0 | 146.3 | 406.5 | 243.9 | 243.9 | 130.1 | 130.1 | 130.1 | 130.1 |
|  |  | Observed | 149 | 81 | 475 | 187 | 240 | 217 | 32 | 148 | 97 |
|  |  | *Wi* | 2.29 | 0.55 | 1.17 | 0.77 | 0.98 | 1.67 | 0.25 | 1.14 | 0.75 |
|  |  | *Bi* | 0.24 | 0.06 | 0.12 | 0.08 | 0.10 | 0.17 | 0.03 | 0.12 | 0.08 |
|  | Socializing | Expected | 31.8 | 71.6 | 199 | 119.4 | 119.4 | 63.7 | 63.7 | 63.7 | 63.7 |
|  |  | Observed | 52 | 44 | 189 | 36 | 175 | 128 | 19 | 87 | 66 |
|  |  | *Wi* | 1.63 | 0.61 | 0.95 | 0.30 | 1.47 | 2.01 | 0.30 | 1.37 | 1.04 |
|  |  | *Bi* | 0.17 | 0.06 | 0.10 | 0.03 | 0.15 | 0.21 | 0.03 | 0.14 | 0.11 |
|  | Overall | Expected | 329.3 | 740.9 | 2058 | 1234.8 | 1234.8 | 658.6 | 658.6 | 658.6 | 658.6 |
|  |  | Observed | 695 | 307 | 2361 | 589 | 1553 | 1062 | 268 | 858 | 539 |
|  |  | *Wi* | 2.11 | 0.41 | 1.15 | 0.48 | 1.26 | 1.61 | 0.41 | 1.30 | 0.82 |
|  |  | *Bi* | 0.22 | 0.04 | 0.12 | 0.05 | 0.13 | 0.17 | 0.04 | 0.14 | 0.09 |
| Dry | Foraging | Expected | 15.6 | 35.0 | 97.3 | 58.4 | 58.4 | 31.1 | 31.1 | 31.1 | 31.1 |
|  |  | Observed | 105 | 18 | 66 | 13 | 28 | 27 | 6 | 33 | 93 |
|  |  | *Wi* | 6.75 | 0.51 | 0.68 | 0.22 | 0.48 | 0.87 | 0.19 | 1.06 | 2.99 |
|  |  | *Bi* | 0.49 | 0.04 | 0.05 | 0.02 | 0.03 | 0.06 | 0.01 | 0.08 | 0.22 |
|  | Resting | Expected | 52 | 117 | 325 | 195 | 195 | 104 | 104 | 104 | 104 |
|  |  | Observed | 282 | 109 | 389 | 55 | 224 | 86 | 6 | 99 | 50 |
|  |  | *Wi* | 5.42 | 0.93 | 1.20 | 0.28 | 1.15 | 0.83 | 0.06 | 0.95 | 0.48 |
|  |  | *Bi* | 0.48 | 0.08 | 0.11 | 0.02 | 0.10 | 0.07 | 0.01 | 0.08 | 0.04 |
|  | Traveling | Expected | 24.8 | 55.7 | 154.8 | 92.9 | 92.9 | 49.5 | 49.5 | 49.5 | 49.5 |
|  |  | Observed | 108 | 30 | 277 | 26 | 83 | 24 | 0 | 38 | 26 |
|  |  | *Wi* | 4.36 | 0.54 | 1.79 | 0.28 | 0.89 | 0.48 | 0 | 0.77 | 0.67 |
|  |  | *Bi* | 0.45 | 0.06 | 0.18 | 0.03 | 0.09 | 0.05 | 0 | 0.08 | 0.07 |
|  | Socializing | Expected | 10.2 | 23.0 | 63.8 | 38.3 | 38.3 | 20.4 | 20.4 | 20.4 | 20.4 |
|  |  | Observed | 45 | 14 | 26 | 7 | 37 | 18 | 36 | 46 | 26 |
|  |  | *Wi* | 4.41 | 0.61 | 0.41 | 0.18 | 0.97 | 0.88 | 1.76 | 2.25 | 1.27 |
|  |  | *Bi* | 0.35 | 0.05 | 0.03 | 0.01 | 0.08 | 0.07 | 0.14 | 0.18 | 0.10 |
|  | Overall | Expected  Observed | 102.5 | 230.7 | 640.8 | 384.5 | 384.5 | 205.0 | 205.0 | 205.0 | 205.04 |
|  |  |  | 540 | 171 | 758 | 101 | 372 | 155 | 48 | 216 | 202 |
|  | | *Wi* | 5.27 | 0.74 | 1.18 | 0.26 | 0.97 | 0.76 | 0.23 | 1.05 | 0.99 |
|  |  | *Bi* | 0.46 | 0.06 | 0.10 | 0.02 | 0.08 | 0.07 | 0.02 | 0.09 | 0.09 |

From Manly *et al*. (2002).

Statistical significance of selection ratios was determined using Bonferroni adjusted 95% confidence intervals of the standardized residuals (Byers *et al*. 1984; Manly *et al*. 2002; Neu *et al*. 1974). All selection ratios were significant except for those highlighted in gray. Wet season = March–October; dry season = November–February; full year = April 2012–March 2013. Overall = aggregate of 15-min scans. MF = mature forest; RVF = riverine forest; SF = secondary forest; YSF = young secondary forest; F3 = fallow stage 3; F2 = fallow stage 2; F1 = fallow stage 1; CAFÉ = coffee plantation; CF = cultivated field.
